# The Pork Worm

**Published:** 1873-07

**Authors:** 


### THE PORK WORM

is found almost exclusively in hog's flesh, not in healthy, well fed pigs; it is found in one animal in fifty slaughtered for the market. It will live for several days in a cold below zero. They can be boiled in meat for twenty minutes and still live; but a heat of one hundred and seventy-five degrees—a heat that will brown a steak—kills them; hence pork, in all its forms, should be well cooked, through and through. Those who suffer most from the pork worm are Germans, who have a taste for eating it raw, or uncooked, as in the form of sausages and smoked ham. Americans abominate uncooked pork, hence do not suffer from trichinal disease. The worm is found in diseased pork with a little covering about it like the "soft" shell of a turtle's egg, but when it is swallowed into the stomach its juices dissolve this shell, and out jumps the little rascal like a chicken out of an egg. As soon as liberated the worm begins to bore a hole in the stomach, and makes its way on, and on, and on until it gets into the solid muscles or flesh of the arms, shoulders, hams and other parts, where it is safe, begetting its kind with amazing rapidity. In February, 1864, over eighty-five thousand of these mischievous creatures were found in a piece of pork measuring an inch each way—a cubic inch of flesh—by Professor Dalton, of New York.

A heat of one hundred and seventy-five degrees dissolves, melts the covering of the worm, as does the gastric juice, and then destroys it. Boiling water measures two hundred and twelve degrees. Fried pork and roast pork require a greater heat than this; hence, when pork is boiled it should be boiled long enough to be boiled through and through ; it should be in boiling water for at least half an hour. The best and safest plan would be to avoid boiled pork altogether ; let it be broiled, roasted or fried.

If pigs are well fed, and kept clean and healthy, their food will be as safe and as nutritious as beef, and even more suitable for cold climates, having a larger amount of carbon. Millions of people in the south and west eat pork in some form almost every day of their lives, sometimes two or three times a day, for weeks together, especially in Kentucky and Tennessee, where the people will compare favorably in size, and strength, and endurance with any other people.

Moses forbade the use of pork, and the Jews are to this day the healthiest people in the world ; but the two facts have no necessary connection. The use of pork flesh was orbidden, as a dividing link between the Israelites and idolaters; it had a religious and not a physical bearing.

Seventy-five of these worms placed in a line make an inch. They were first described by Paget in 1835, and were after found in the muscles in the dissecting room, but were never regarded as a cause of disease until 1860, when Professor Zenken, of Dresden, traced the origin of the disease in a young girl who had been ill several weeks after eating some ham and sausage. She suffered from diarrhœa, weakness, fever, sleeplessness, tenderness over the stomach, great pain in the voluntary muscles—those which we can move at pleasure, and where alone they settle—swollen feet and death, which often takes place within twenty days after the diseased meat is eaten.

Hog cholera is believed to be the result of the presence of the pork worm. When once infected a man is liable to have the disease at any time; it is not necessarily fatal, but there is no known cure.

